# Age Differences in Discrete Emotional States During Risk Taking

**DOI:** 10.1093/geroni/igaa057.1832

**Published:** 2020-12-16

**Authors:** Nathaniel Young, Joseph Mikels

**Affiliations:** DePaul University, Chicago, Illinois, United States

## Abstract

Emotions often guide risk-taking. For example, anger tends to lead to increased risk-taking. However, older and younger adults differ in their emotional experiences: older adults tend to report more positive emotions, fewer experiences of anger, and relatively similar or increased experiences of sadness relative to younger adults. As such, differences in emotional experience may manifest in the integral emotional responses of older and younger adults as they take risks. The current work examined the discrete integral emotional responses of older and younger adults as they completed the Balloon Analogue Risk Task (BART). For the BART, participants completed 40 trials. Prior to each trial, participants reported how much anger, sadness, contentment, and excitement they felt. The results indicate that younger adults experienced more anger and less contentment than older adults in response to the BART. Importantly though, age differences also emerged in how discrete emotions predicted subsequent risk-taking.

